# The Comparison of the Effect of Transactional Model-Based Teaching and Ordinary Education Curriculum- based Teaching Programs on Stress Management among Teachers

**DOI:** 10.5539/gjhs.v6n3p241

**Published:** 2014-04-09

**Authors:** Seyed Saeed Mazloomy Mahmoodabad, Maryam Mohammadi, Davood Shojaei Zadeh, Abolfazl Barkhordari, Fatemeh Hosaini, Mohammad Hossain Kaveh, Amal Saki Malehi, Mohammadkazem Rahiminegad

**Affiliations:** 1 Department of Health Sciences, School of Health, Yazd Shahid Sadoughi University of Medical Sciences, Yazd, Iran; 2 Department of Health Sciences, School of Health, Tehran University of Medical Sciences, Tehran, Iran; 3 Department of Health Sciences, School of Health, Shiraz University of medical sciences, Shiraz, Iran; 4 Department of Health Sciences, School of Health, Jondishapoor University of Medical Sciences, Ahvaz, Iran; 5 Department of Education and Training Office, Yazd, Iran

**Keywords:** transactional model, education program, stress management

## Abstract

**Background and Objectives:**

Regarding the effect of teachers’ stress on teaching and learning processes, the researchers decided to provide a stress management program based on Transactional Model to solve this teachers’ problems. Thus, this study is going to investigate the effect of Transactional Model- based Teaching and the Ordinary Education Curriculum- based Teaching programs on Yazd teachers.

**Methods:**

The study was a semi- experimental one. The sample population (200 people) was selected using categorized method. The data were collected via PSS Questionnaire and a questionnaire which its validity and reliability had been proved. Eight teaching sessions were hold for 60-90 min. Evaluation was performed in three steps. The data were described and analyzed using SPSS software version 15. Value of P<0.05 was considered as significant.

**Results:**

The participants were 200 people of Yazd teachers of primary schools. Mean age of group 1 and 2 was 42.05±5.69 and 41.25±5.89 respectively. Independent T- Test indicated a significant mean score (p=0.000) due to perceived stress of interference groups in post interference step and follow-up one respectively.

**Conclusion:**

Results showed a decreasing effect of both programs, but the Transactional Model- based interference indicated to decrease stress more than the other.

## 1. Introduction

Education system is regarded as one of the most important, complicated and large–scale organizations of the society. Most of these organization goals are realized by teachers –as an important core of the organization. Being neglected teachers’ mental health affected students and educational system badly (Shahmansuri et al., 2013; Bush, 2007). Stress or tension -one of the important issues of mental health- imposes a great damage on teaching and learning processes. It also results in physical, emotional and attitude burnout (Shimazu et al.,2003). Stress is defined by Lazarus and Folkman as individual response against the threatening environment and against her/his abilities, sources and health (Lundberg, 2005). The teachers’ responsibilities of students’ improvement, happiness and activities are particular ones. Therefore, teachers’ stress is separated from other jobs’ stress (Tolbert et al., 2007). Since the emotional and physical pressures due to stress are regarded as harmful and undesirable, the individuals are encouraged to do something to decrease the stress (Sarafino, 1999). In fact, a set of activities done to decrease the stress is called coping. According to Folkman and Lazarus, coping consists of individual cognitive and behavioral attempts which are going to decrease the stresses due to internal or external needs. According to individual perception, these needs go beyond the individual sources available (Lazarus & Folkman, 1984)

Studies showed an important role of transactional model for reducing stress (Yong Wah, 2010; Akihito, 2003; Lauga, 2008; Sharron, 2011). But in regard to our knowledge, this study was evaluating all components of transactional model for the first time. Besides, in this study was compared routine method and thransactional model for teachers.

Most cure professions including nursing, medicine and other human services ones are considered stressful (Russel, 1997). Teaching is also particular in view of the responsibility to health, bliss and activities of the students. Teachers are responsible for promotion of knowledge, pedagogy of students and creating discipline so, teachers, stress is of different type.

Although many teachers are fond of their job and experience little strain, several surveys have documented that up to a third of the teachers consider teaching as highly stressful (Farsani, 2012)

Despite the researches indicating a high value of stress among Iranian teachers and their sources (Allahverdipou, 2005; Karbasi, 2000; Ahmady et al., 2007; Kyriacou et al.,1987) unfortunately, little has been done to cope with them. In addition, teaching the health using cultural and social patterns plays an important role in prevention and controlling diseases and health problems. Lazarus and Folkman Transactional Model is considered as one of the most completed models concerning with stress which combine both cognitive and coping processes (WHO, 1993; Folkman & Lazarus, 1990; Khan et al., 1991). Thus, We compare the efficacy of interventions based on transactional model of stress and coping with stress for reducing of stress in teacher of Yazd city and this study was aimed to answer to these questions as follows:

1) Is education based- routine program useful for reducing stress in teachers?
2) Is education based- transactional model program useful for reducing stress in teachers?
3) Is difference in stress level after the intervention program in the intervention group based on transactional model and routine method?
4) Does the effectiveness of intervention programs transtheorical model is better than routine method?

### 1.1 Transactional Model of Stress and Coping

Transactional Model of Stress and the approaches of coping with it, are regarded as the framework to evaluate the coping with stress process. The model is consists of a perceptual theory of stress containing the following components:

Primary Appraisal: this concern with the importance of stressor event in first glance of individual, and whether it is a positive, controllable, problematic or unrelated one (Folkman,2000)

Secondary Appraisal: It is a type which investigates coping sources (Cohen et al.,1984) considering his/her possibilities; the individual chooses a solution to face threat or challenge.

Coping Efforts: The efforts in Transactional Model consist of two aspects:

-Problem Management: In this step it is suggested to solve the problem using problem- based coping to change stressor condition.
-Emotional Regulation: The object of the strategy is to change individual thought and emotion against stressor conditions (Carver, 2001).

Meaning-based Coping: The approach aims positive emotion and includes positive reappraisal, acceptance and using religion power. Stressor conditions are interpreted using meaning-based approach.

Moderators: Moderators are divided as follows:

-Dispositional Coping Style: Despite of the condition-specific coping, coping styles are regarded as stable characteristic representing general tendencies toward interpretation and reaction to stress. The styles may sometimes last a long time and vary due to different individual and characters (Lazarus, 1993).
-Social supporting: Social supporting has been presented in form of theory in different ways. Some believe in objective and subjective dimensions, while others emphasize on non-subjective aspects including dependence and belonging emotions, or qualitative aspects, for example, subjective appraisals (Cohen et al.,1984). Social supporting, influencing some of the key processes of Transactional Model may positively affect individual approach due to stressor situations (Heitzman et al., 1998).

### 1.2 The Result of Coping

The results show the individual adaptation against stressor factor when environment appraisals done.

## 2. Methods

### 2.1 Participants and Study Design

This semi-experimental research was conducted to determine the effect of Ordinary Education Curriculum- based Teaching and Lazarus and Folkman Transactional Model- based Teaching programs on decreasing the stress and its factors related. The sample population concluded 200 teacher of Yazd, a big city of Iran (Ghasemi, 2011; Jöreskog, 1993). One hundred people as interference group 1 (following Ordinary Curriculum-based teaching) and 100 people as group 2 (following Transactional Model-based teaching) were chosen in a categorical method.

Teachers were divided into two classes based on Girl´s School and Boy´s School. Then, one hundred teachers randomly selected using equal allocation of each class. Data were collected using PSS questionnaire for stress (Cohen,1983). PSS questions about was designed based on a person’s thoughts and feelings that have been proposed in the past month and responders explain their opinion about being uncontrollable, unpredictable and challenging time of his life. In addition, the scale has a number of direct questions about levels of stress. There are 14 questions and 10 questions on this scale version that the 14-item version was used in this study.

The constructs of Transactional Model were measured by the questionnaire provide. The questionnaire concluded demographic data and 62 questions measuring model constructs, which their validity and reliability were evaluated by researchers. Content validity, and CVR=0.85 methods were used to study validity and internal adaptation respectively. Criterion validity with Pierson correlation showed a significant estimation power (r= 0.75) i.e. (p<0.001). Coronbach alpha coefficient value (0.87) indicated a good reliability. Entering the study criteria were as follows: being a teacher of primary schools, no previous participating in stress management course and regular attending in educational program presented. In addition to taking participants’ consent, they were assured that data will be patented. After the questionnaires were planned and confirmed by experts, the project and its object were introduced to Education Administration of Yazd. Then, the implementation license was issued by the officials.

### 2.2 Intervention Program

Eight sessions of 60-90 min were hold observing syllabuses and contents specified. The interference with Transactional Model group programs concluded lecture, panel discussion and teaching sources including teaching package provided based on Transactional Model, power point, pamphlet, CD of muscle relieving (as homework).

### 2.3 Content of Education for Groups

Content of education - based on education and training (group 1)

Section 1: definition of Stress
Section2: stages of stress
Section3: causes of stress
Section4: Stress symptoms
Section5: Burnout
Section6: Ways decrease of Stress
Section7: Coping Strategies
Section8: recommendation for preventive of burnout in teachers

Content of education based on Transactional Model (group 2)

Section 1: Overview of program and transactional model
Section2: Introduction to stress and its role
Section3: emotional regulation (emotion-focused coping)
Section4: Problem management (problem-focused coping)
Section5: Self-efficacy and self-confidence
Section6: Social relationship
Section7: Meaning-based coping
Section8: Time management and physical ways for prevention of stress

Evaluation was done in three steps: before the period began, immediately after it’s finishing, and one month later it finished, in which instrument questions were answered by participants of both groups.

### 2.4 Data Analysis

The K-S test was used to describe and analyze the data and determine whether they were distributed normally or not. The value of p<0.05 was considered as significant. SPSS software version 15 was used to analyze the data.

## 3. Results

### 3.1 Demographics

Mean age of interference group 1 and 2 were 42.05±5.69 and 41.25±5.89 respectively. Most of the participants were married, enjoying more than 20 years work experience. They had been received B.A in Primary Teaching too. The mean stress score showed no significant difference between two groups in pre-interference step, i.e. they were equal.

### 3.2 Explanatory Analyses

**Figure 1:**
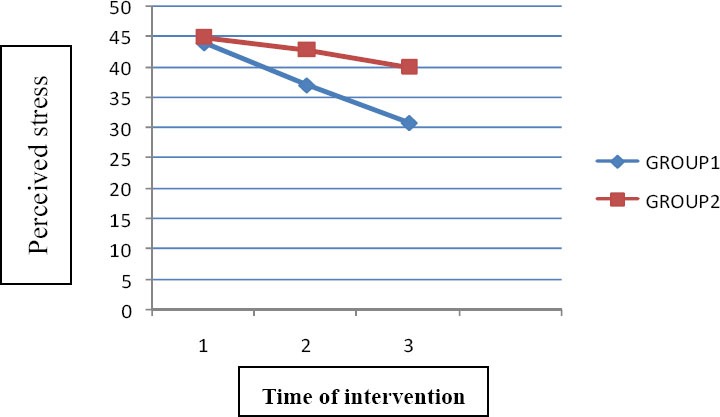
Comparison of perceived stress in pre-test, post- test and follow up

The bilateral variance analysis test results indicated that none of the variables studied had significant effect on perceived stress scores in pre-test and follow-up steps. Stress mean scores of group 1 in pre- interference step i.e. 45.49±5.15 decreased to 40±6.28 in follow-up step while the mean in group 2 was 44.71±0.48 and 30.76±0.55 in pre-interference and follow-up steps respectively (Figure 1).

The independent t-test results indicated a significant difference in mean scores due to perceived stress in interference groups in post- interference and follow-up steps (P<0.05). The study of mean scores difference in between the groups represented a significant value in interference group 2 concerning all constructs in follow-up step, confirming long-term effects of the program on stress value. This mean indicated a significant value in interference group 1 in pre-test and post-test steps. The effects of teaching decreased after taking post-test, but the difference was not significant (Table 1).

**Table 1:** Results of pair comparison of pre-test, post- test and follow up

Construct	Time	Group 1		Group 2	
		Significant Level	Mean Difference	Significant Level	Mean Difference
Primary appraisal	Pre-test, Post-test	1	0.000	1.7	0.021
	Pre-test, Follow up	1.083	0.642	3.79	0.000
	Post-test, Follow up	0.830	0.642	2.09	0.006
Secondary Appraisal	Pre-test, Post-test	0.650	0.294	1.59	0.002
	Pre-test, Follow up	0.630	0.392	2.43	0.000
	Post-test, Follow up	1.280	0.052	0.84	0.056
Problem Management	Pre-test, Post-test	5.210	0.000	0.14	0.752
	Pre-test, Follow up	5.830	0.000	3.15	0.000
	Post-test, Follow up	0.620	0.547	3.29	0.000
Emotional Regulation	Pre-test, Post-test	0.260	0.60	0.71	0.60
	Pre-test, Follow up	1.050	0.020	3.15	0.002
	Post-test, Follow up	1.310	0.007	1.62	0.007
meaning-based coping	Pre-test, Post-test	1.630	0.069	0.77	0.069
	Pre-test, Follow up	1.650	0.054	1.19	0.014
	Post-test, Follow up	2.020	0.22	0.42	0.22
Adaptation	Pre-test, Post-test	2.050	0.002	0.88	0.12
	Pre-test, Follow up	0.980	0.101	1.53	0.001
	Post-test, Follow up	1.070	0.087	0.65	0.087
Moderators	Pre-test, Post-test	0.790	0.197	2.81	0.000
	Pre-test, Follow up	0.750	0.097	0.52	0.097
	Post-test, Follow up	0.040	0.950	3.33	0.000
Perceived Stress	Pre-test, Post-test	2.250	0.14	2.33	0.03
	Pre-test, Follow up	5.490	0.025	4.95	0.000
	Post-test, Follow up	3.240	0.12	2.62	0.005

These means were compared using independent t-test, statistic analysis method and repeated variance to study the effect of teaching on Transactional Model constructs, and possibly difference between the model constructs mean scores in pre-test, post-test and follow-up steps. Variance analysis results, containing repeated values, indicated that the difference between pre-test, post-test and follow-up mean scores in both groups were generally significant. Mauchely’s test results confirmed “equal co-variances between dependant variables” presupposition, and the values (Table 2).

**Table 2:** Results of repeated measures variance analysis on Transactional Model constructs of pre-test, post test and follow up

Constructs	pre-test				T-test	post test				T-test	follow up				T-test	repeated measures
	Group1		Group2			Group1		Group2			Group1		Group2			
	M	SD	M	SD		M	SD	M	SD		M	SD	M	SD		
Stress	45.49	6.28	44.71	0.48	P=0.132	43.24	6.62	37.38	0.53	P=0.000	40.00	5.15	30.76	0.55	P=0.000	P=0.000
					t=0.325					t=8.05					t=6.327	F=32.127
					F=0.973					F=5.27					F=0.265
Primary appraisal	19.67	3.05	19.52	0.49	P=0.089	20.67	5.91	20.22	0.43	P=0.005	21.50	4.44	23.31	0.47	P=0.000	P=0.000
					t=1.70					t=2.87					t=6.241	F=15.611
					F=0.69					F=0.077					F=0.687
Secondary Appraisal	17.77	5.40	18.20	0.30	P=0.342	18.42	4.43	20.79	0.29	P=0.000	17.14	4.68	20.63	0.22	P=0.000	P=0.000
					t=0.363					t=13.398					t=19.799	F=96.837
					F=0.833					F=43.47					F=3.763
Problem Management	35.61	4.03	35.35	0.48	P=0.679	40.83	4.03	35.21	0.55	P=0.000	41.44	6.01	44.50	0.32	P=0.000	P=0.000
					t=0.414					t=13.148					t=18.992	F=88.816
					F=1.55					F=24.64					F=20.36
Emotional Regulation	18.23	3.00	18.13	0.39	P=0.920	17.79	3.71	18.47	0.31	P=0.000	19.28	3.15	21.03	0.22	P=0.000	P=0.000
					t=0.101					t=9.276					t=10.951	F=197.00
					F=4.53					F=4.642					F=6.870
meaning-based coping	18.71	3.43	19.24	0.38	P=0.304	20.34	8.00	23.41	0.38	P=0.000	21.36	3.62	24.83	0.34	P=0.000	P=0.000
					t=1.022					t=13.435					t=31.07	F=171.252
					F=0.682					F=45.51					F=0.016
Moderator	11.25	3.03	14.02	0.45	P=0.000	12.04	5.17	15.14	0.34	P=0.000	12.00	3.39	16.49	0.32	P=0.000	P=0.000
					t=0.853					t=8.088					t=5.644	F=40.99
					F=0.034					F=0.262					F=0.336
Adaptation	27.91	4.51	28.14	0.43	P=0.161	25.86	4.86	28.33	0.48	P=0.000	26.93	4.06	33.66	0.41	P=0.000	P=0.000
					T=1.406					t=11.165					t=11.165	F=95.856
					F=14.87					F=16.35					F=16.35

## 4. Discussion

The object of this semi-experimental project was to measure the effect of Transactional Model on decreasing stress among teachers in comparison with Ordinary Education Curriculum. The research attempted to measure the effect of a teaching package –provided based on Transactional Model- on stress management skill in comparison with Ordinary Education Curriculum.

The findings indicated that perceived stress mean scores of interference group with Transactional Model significantly were decreased in post-interference and follow-up steps in relation to another group in interference step. The finding evidenced decreasing effect of the program on stress. The results of this study comply with Lauga et al research demonstrating the decreasing effect of Transactional Model on teachers, tension and mental fatigue (Lauga, 2008). Also, Akihito et al showed in their study that using an intensive program for a certain group improves coping skills, social supporting and decreases stress reactions (Akihito et al.,2003).

In this research, the effect of Model-based program in follow-up step was more than post- interference step. The possible reason was that participants had a chance to apply the skills learnt in real life situations and fix them in their routine activities (Meichenbam, 2007).

Besides, Sharron and colleagues showed the efficacy of a brief cognitive-behavioral program for reducing the work-related stress of teachers (Sharron, 2011).

This was probably because of using both lecture and questions and answers methods as well as training materials (audio-visual). It tends to have lasting effects training. Besides, using group discussion was caused the development of intellectual and cognitive skills of individuals as well as a lasting education.

In addition, coping with stress behaviors (stress management, emotional regulation, meaning- base coping and moderators) were studied in the research. According to findings, mean scores of interference group increased in post-interference step and the difference in relation to pre-interference step of the same group and control group in post-interference step was significant. This finding complies with Rezaee research demonstrating the effect of teaching stress on nurses’ stress value (Rezaei et al.,2006).

Akihito and coworkers reported that a stress management program might be effective in enhancing social support and decreasing stress responses in teachers (YongWah et al.,2010)

Perhaps it is for this reason that the model has a certain structure and it combine cognitive and behavior processes and it makes the program more efficient than other programs.

Generally, the results demonstrated that Transactional Model- based interference program decreased teachers’ stress more than Ordinary Program. This complied with Yong Wah et al research showing the decreasing effect of Transactional Model- based program on stress (YongWah et al.,2010).

## 5. Conclusion

Totally, the program provided a chance for participants to learn some efficient techniques which complied with their work conditions familiarizing teachers with stress nature and effects. It also reminded them the esoteric and social skills which could use in different situations. Being hopefully effective the Transactional Model- based program in improving teachers’ mental health and decreasing stress among them, it is recommended to provide similar teaching programs for other stressor jobs, for example nursing and medicine, or implement the present program in other education environments.
